# Analysis of methylation and mRNA expression status of*FADD* and*FAS* genes in patients with oral squamous cell carcinoma

**DOI:** 10.4317/medoral.19805

**Published:** 2014-08-17

**Authors:** Eshaghali Saberi, Dor M. Kordi-Tamandani, Sara Jamali, Mohammad A. Rigi-Ladez, Arsalan Augend

**Affiliations:** 1 Department of Endodontic, Dental school, Zahedan University of Medical Sciences, Zahedan, Iran; 2 Department of Biology, University of Sistan and Baluchestan, Zahedan, Iran; 3 Genetic of noncommunicable diseases research center, Zahedan University of Medical Sciences, Zahedan, Iran; 4 Dental Research Center Zahedan University of Medical Sciences, Zahedan, Iran; 5 Department of Maxillofacial Surgery, Dental School, Zahedan University of Medical Sciences,zahedan,Iran

## Abstract

Background: Apoptosis is an important mechanism that is responsible for the physiological deletion of harmful, damaged, or unwanted cells. Changed expression of apoptosis-related genes may lead to abnormal cell proliferation and finally to tumorigenesis. Our aims were to analyze the promoter methylation and gene expression profiles of *FADD* and *FAS* genes in risk of OSCC. Material and Methods: we analyze the promoter methylation status of *FADD* and *FAS* genes using Methylation - Specific PCR (MSP) in 86 OSCC tissues were kept in paraffin and 68 normal oral tissues applied as control. Also, *FADD* and *FAS* genes expression were analyzed in 19 cases and 20 normal specimens by Real-Time Reverse-Transcripts PCR. Results: Aberrant promoter methylation of *FADD* and *FAS* genes were detected in 12.79 % (11 of 86) and 60.46 % (52 of 86) of the OSCC cases, respectively, with a significant difference between cases and healthy controls for both *FADD* and *FAS* genes (*P*<0.001). The gene expression analysis showed statistically significant difference between cases and healthy controls for both *FADD* (*p*<0.02) and *FAS* (*p*<0.007) genes. Conclusions: To the best our knowledge, the data of this study are the first report regarding, the effect of promoter hypermethylation of the *FADD* and *FAS* genes in development of OSCC. To confirm the data, it is recommended doing further study in large sample sizes in various genetic populations.

** Key words:**OSCC, FADD, FAS, DNA methylation, gene expression.

## Introduction

Head and neck cancer holds the sixth place in the cancer incidence ranking worldwide, influencing almost 650,000 people and causing nearly 350,000 cancer deaths each year (1,2). Among all of the head and neck cancers, Oral Squamous Cell Carcinoma (OSCC) is the most prevalent malignant epithelial neoplasm influencing the oral cavity (3).

The incidence and development of OSCC are multi stage processes, which arises through a collection of genetic and epigenetic variations (4). DNA methylation, as a key epigenetic variation is necessary for normal differentiation and development. The aberrant DNA promoter methylation that influences gene expression is a common feature of many human cancers (5). With respect to OSCC development, recent works demonstrated that hypermethylation of CpG islands of genes that are implicated in apoptosis, DNA-repair, cell-cell adhesion, and cell cycle regulation plays a vital role in cancer progression (6-9).

Apoptosis or programmed cell death deals with a significant task in the maintenance of cellular homeostasis. The inactivation of apoptosis related genes may lead to unusual cell proliferation and tumorgenesis (10,11). Generally, apoptosis is regulated by two major pathways: the receptor-mediated and the intrinsic (mitochondrial) pathways (12). Fas (CD95/Apo1) are a cell surface receptor that belongs to tumor necrosis factor receptor (TNF-R) family. Physiologically, it is expressed in various tissues such as lymph nodes, spleen and on mature hematologic cells (13,14).

Fas Ligand (FasL) is a homotrimeric protein act as a ligand for Fas receptor and causes its oligomerization. This process vast through the death domain (DD) and Fasassociated death domain (*FADD*). The N-terminal region of *FADD* which comprises DED (Death Effector Domain) motif binds to a homologous motif in Procaspase-8. Caspase-8 activates caspase-3 and -7 that mediate cell death. Furthermore, it cleaves Bid to generate truncated Bid (tBid) which translocates to the mitochondria and triggers the mitochondrial apoptotic pathway (15,16). Aberrant promoter methylation of FAS and FADD gene were exposed in different types of human cancers (17). Further, Fas is expressed in high quantities in lower stage of OSCC and a high incidence of *FADD* expression was significantly correlated with lymph node metastasis of SCCs (18,19). The data have been reported rarely, regarding to the status of the methylation and expression profile of *FADD* and *FAS* genes in OSCC tissues. Hence, the present study is trying to highlight the expression and methylation profile of *FADD* and *FAS* genes in patients with OSCC.

## Material and Methods

-Samples and DNA preparation

This study involves 86 tumor specimens of OSCC (mean age 54.37 ±14) that had been fixed in Paraffin and 68 oral mucosa biopsies as controls (mean age 41±14) were collected during surgical resections of oral region squamous cell of patient’s (gingival aria) without a history of OSCC who were referred to Periodontics Department, after explanation of study purpose and signing of consent form.

Clinic pathological data of the patients and the controls such as age, sex, and clinical stage are shown in  Table 1 and  Table 2. Genomic DNA was isolated from tumor and healthy tissue samples using QIAamp DNA extraction kit (Cat. No. 56404, Qiagen) according to the manufacturer’s instructions and then its quality was estimated by Spectrophotometer.

**Table 1:**
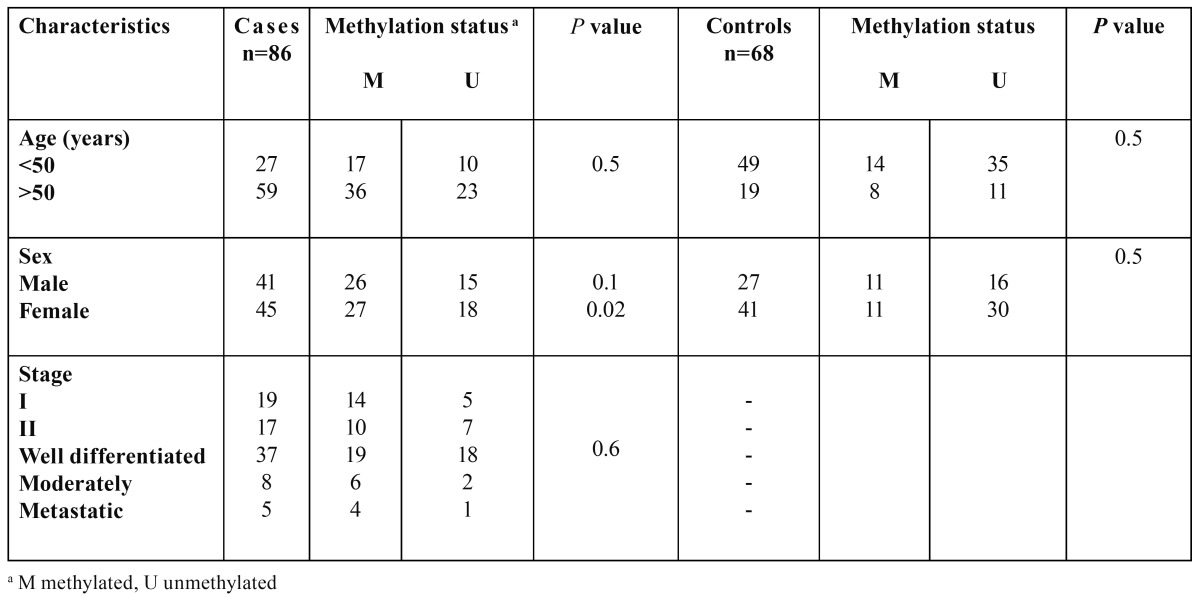
Association between Fas gene promoter methylation and clinicocopathological parameters in patients with OSCC and health controls.

**Table 2:**
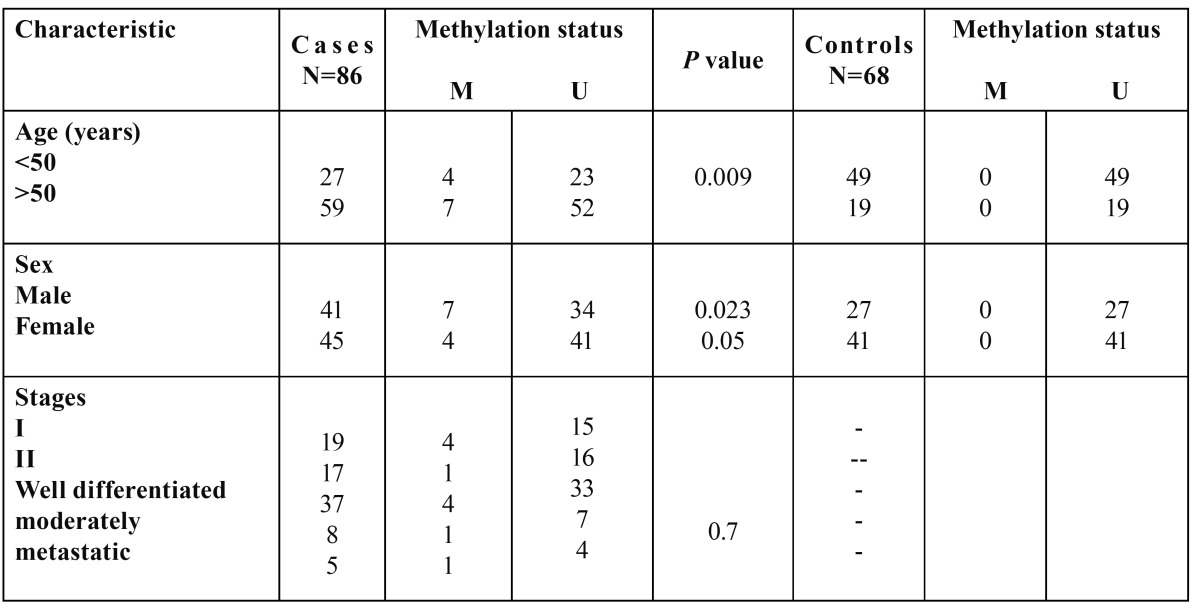
Association between *FADD* gene promoter methylation and clinicocopathological parameters in patients with OSCC and health controls.

-Methylation-Specific PCR (MSP)

**Figure 1:**
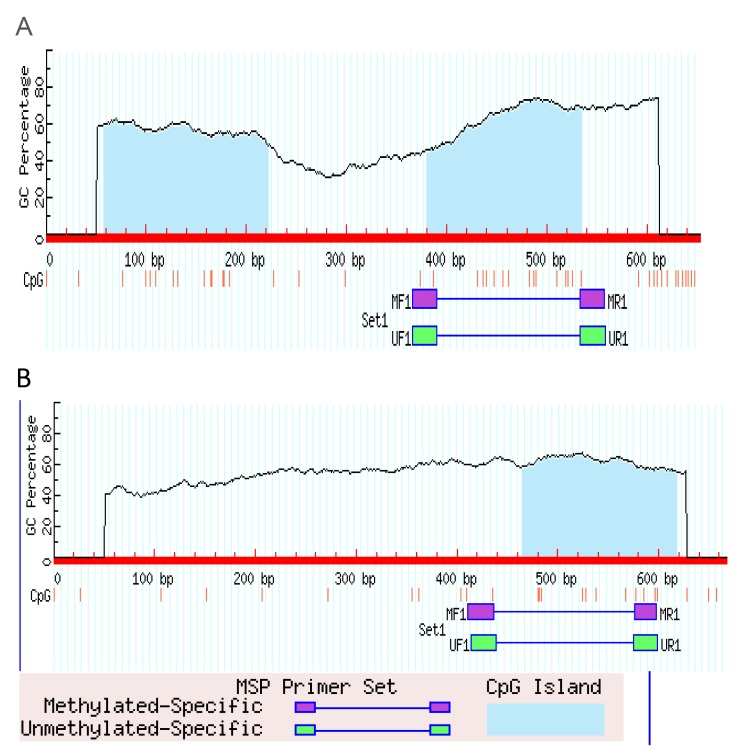
Selcted CPG island for MSP amplification, near to transcription start point A: *FADD*-001 ENST00000301838 and B; *FAS*-003 ENST00000460510.

The process of bisulfit modification of DNA samples was performed as previously described (20). Methylation status of the promoter regions of *FADD* and *FAS* was determined by Methylation-Specific PCR (MSP) using methylated specific and unmethylated specific primers was designed at CpG sites of the promoter region using MatPrime online software (Fig. 1,  Table 3).

**Table 3:**
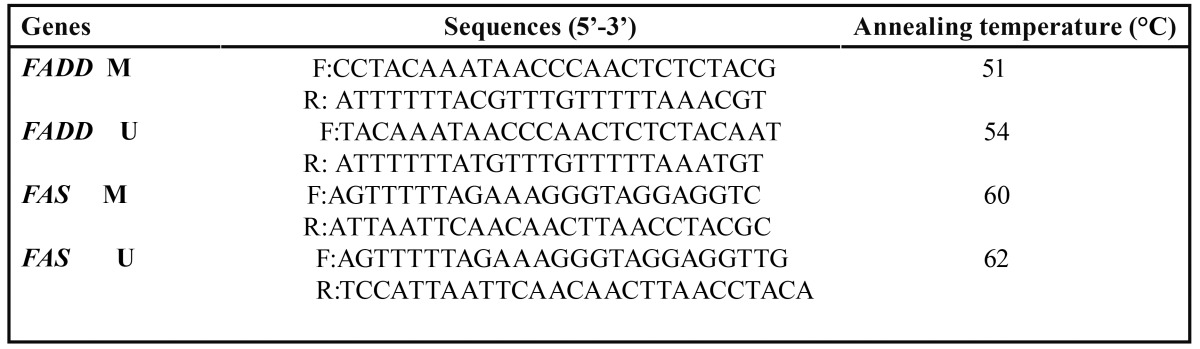
Primer sequences and annealing temperatures.

The PCR reaction mixture included 2 μL of modified template DNA, 0.2 µL of HotStarTaq®, 1 µL of dNTP mix (10 µmol/L), 16.3 µL of RNase free double distilled water, 2.5 µL of 10× buffer, 0.5 µL of each primer (10 µmol/L), and 2 µL of Mg2+ (25 µmol/L) in total (a) volume of 25 µL.

**Figure 2:**
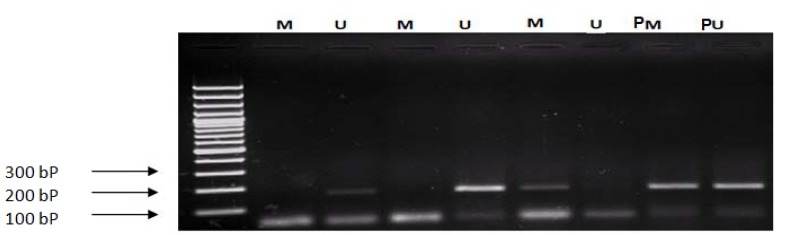
Methylation analysis of *FADD* gene: M: amplified product regoconizse by methylated primer (194 bp) U: amplified product regoconizse by unmethylated primer (192bp); L: ladder 100 bp: PM and PU (Human HCT116 DKO Methylated and Unmethylation DNA, D5014-1,2: Zymo Research California, USA).

**Figure 3:**
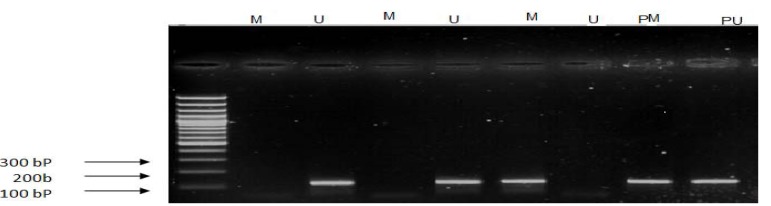
Methylation analysis of *FAS* gen, M: amplified product regoconizse by methylated primer( 160bp), U: amplified product regoconizse by unmethylated primer( 165bp), L: ladder 100 bp.

MSP amplification was performed as follows: 94°C for 10 min; and then 40 cycles consisting of (40 s at 94°C, 30 s at 51°C for *FADD* (M), 54°C (U), and for *FAS* 60°C (M), 62°C (U), 1 min at 72), and a final extension at 72°C for 10 min. PCR products were loaded onto 4% agarose gel and stained with ethidium bromide (Figs. 2,3).

-Gene expression analysis

Total RNA was extracted from OSCC fixed paraffin embedded tissue sections and fresh normal samples that had been taken during various surgical operations in oral region, except of cancerous cases. It were used (Using) the High Pure FFPE RNA Micro Kit) Cat No: 04823125001 (and Cinna Pure RNA Purification Kit) Cat No: PR891620 (respectively, according to the manufacturer’s instructions.

The cDNA Synthesis Kit (Fermentas, Cat No: K1621) was used to reverse-transcribe 1 μg of RNA in a final volume of 20 μl. As an internal standard, RNA18S was used. Real time-PCR of *Fadd* and *Fas* were performed using the primers and annealing temperatures in  Table 4. Cycle threshold (CT) at which the fluorescence for the reaction well crosses was recognized for each gene in all samples and then, normalized CT (CT target gene/CT housekeeping gene) was used for comparison of genes expression between groups.

**Table 4:**
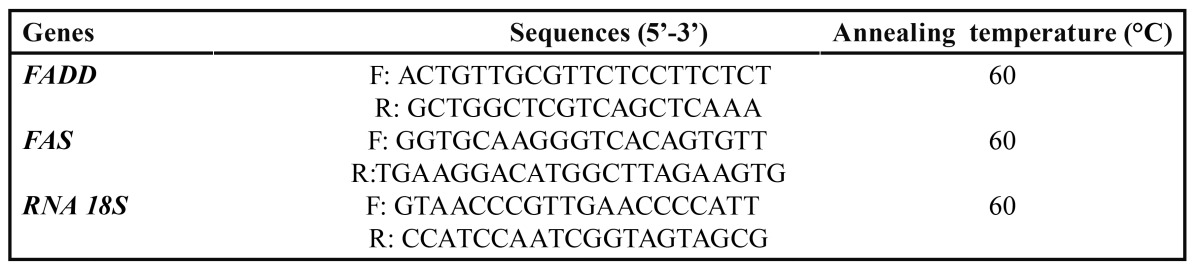
Real-time primer sequences and annealing temperatures.

-Statistical analysis

Data were analyzed using SPSS software. The chi-square test was used for categorical variables. The effect of the methylation of *FADD* and *FAS* genes on the risk of OSCC was detected by estimating odds ratios (OR) and 95% confidence intervals (95% CI) using the binary logistic regression test. Analysis of relative gene expression between patients and controls was done by mann-whitney test. The significance level was set at p≤0.05 for all the tests.

## Results

-Promoter methylation of *FADD* and *FAS*

Promoter methylation status of the *FADD* and *FAS* genes in patients and healthy individuals and their relationship with risk of OSCC is indicated in  Table 5 and  Table 6. As shown, the frequency of methylation status for *FADD* gene was 12.79 % in tumor tissues (11 of 86 cases) and zero for normal mucosa (68). So (On the other side), it was not appeared a significant association between methylation status of *FADD* gene and risk of OSCC. Regarding the *FAS* gene, the amount of methylation was 60.46 % for cases (52 of 86) and 39.54% (22 of 68) for controls that this difference statistically, was significant between groups (*P*< 0.001). In addition, it was appeared a significant association between methylation status of FAS gene and increased risk of OSCC (OR=2.622, 95% CI; 1.18-5.82, *P*< 0.018).

**Table 5:**
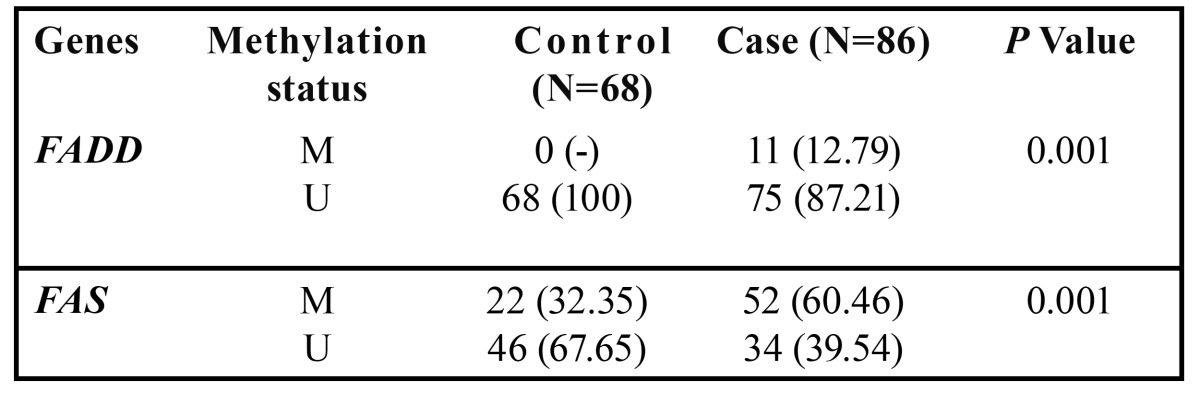
Promoter methylation frequency of *FADD* and *FAS* genes in patients with OSCC and healthy controls.

**Table 6:**
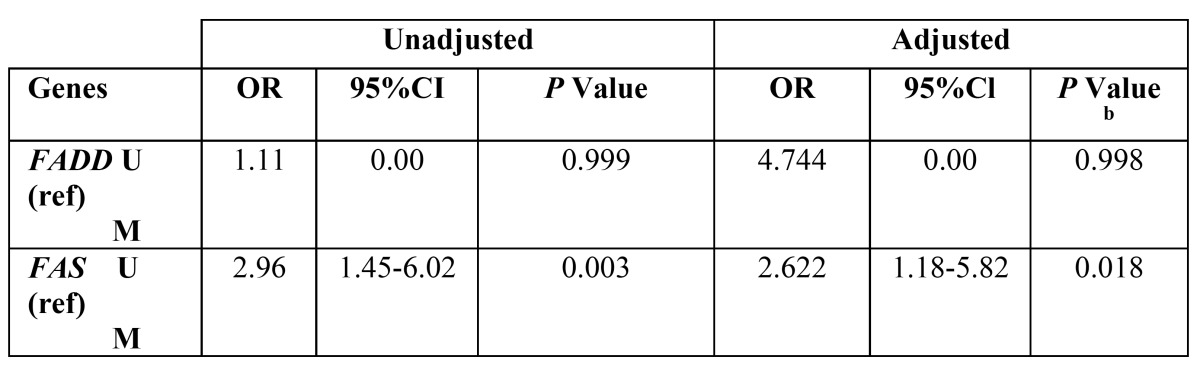
Risk of OSCC based on gene promoter methylation.

-*FADD* and *FAS* mRNA levels

Assessment of relative gene expression was done according to dividing CT target gene to CT housekeeping gene for *FADD* and *FAS* between groups. As shown in  Table 7, the mean of relative expression for *FADD* was 0.9527±0.57 in cases (n=25) and 1.9068 ±0.48 in controls (n=19). The *FAS* outcomes were 0.96±0.58 for cases (n=25) and 1.9926 ±0.36 for controls (n=20). The differences of relative gene expression between patients and healthy individuals were statistically significant for both of them (*P*< 0.0001).

**Table 7:**
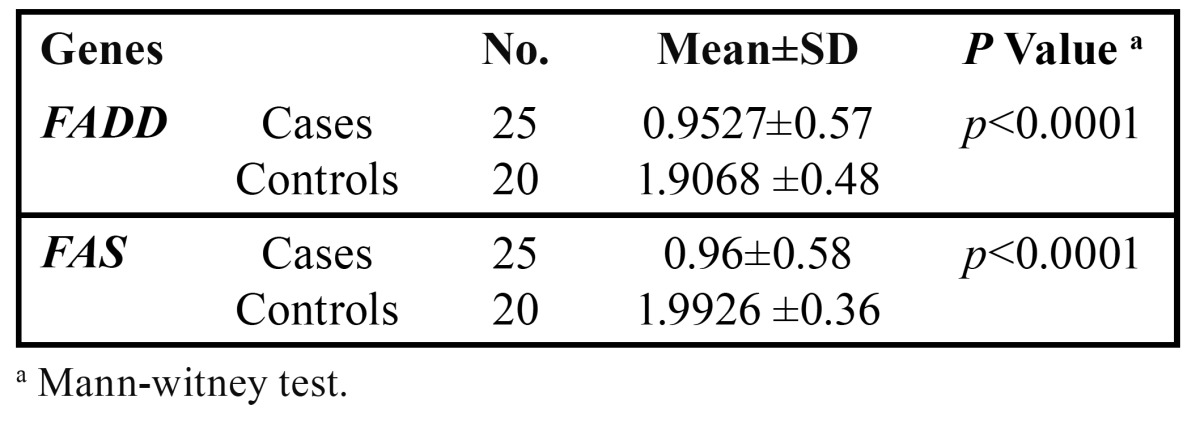
Comparison of relative gene expression for *FADD* and *FAS* genes between patients with OSCC and healthy controls.

## Discussion

Epigenetics is a study of heritable variations that interferes in gene function without modifying the DNA sequences (21). It is responsible for the stable maintenance of a particular gene expression pattern through the cell cycle. The realizing of epigenetic mechanisms, including DNA methylation and chromatin remodeling, have shown a rapid progress in diagnosis and treatment of various diseases (22). These variations may induce gene silencing, imprinting and RNA interference that may lead to unusual modification as tumorigenesis (23). The results of methylation analysis in this study showed statistically, significant difference in amount of promoter methylation status between cases and healthy controls.

In line our results, Li W *et al*., (2011) shown that the rate of *FAS* promoter methylation in bladder urothelial carcinoma samples is higher than normal samples (*p*<0.01) (24). In addition, a vast literatures have been highlighted the aberrant promoter methylation of *FAS* gene in different types of cancers including Lymphomas, CXCA, melanoma, Colon, Prostatic and Lung (25-28).

To play a significant role in down regulation of *FAS* and *FADD* expression in early stage of tumorogenesis. The outcomes of the present gene expression analysis exposed a higher ratio of expression for *FADD* and *FAS* in patients with OSCC than healthy controls.

*FAS* and *FADD* are the most important elements of the apoptotic pathway with role of removing of harmful, damaged, or unwanted cells (29). Some studies have suggested that impairment of *FAS* gene expression links with development of various tumors such as; stomach, esophagus and liver (30-32). Muraki *et al*., (2000) have reported the increased expression of *FAS* gene in lower stage of SCC, it might operate as a controlling factor to promote apoptosis at the first step of disease but, in advanced stage of the disease has been detected to be down-regulated (33,34). The expression of the *FADD* has been found to be linked with non-small cell lung cancer and poor survival in laryngeal carcinoma (35,36). Accordance to our study, Lo Mozio *et al*., (2008) reported that there is a significant difference for expression of FADD gene between OSCC patients and healthy controls (37,38). In summary, this study tried to demonstrate the patterns of *FAS* and *FADD* genes methylation and expression profile in OSCC within a Southeastern Iranian population. The different expression of these genes between ill and normal groups is highlighting their significant role in development of OSCC. Ultimately. It should be mentioned that mathylation could be one of the reasons of gene expression changes. Therefore, we would like to suggest further studies to identify exact molecular process of the disease using advanced molecular techniques such as Micro Array and Meth Light in various and larger genetic populations.
